# Measuring Volumetric Changes of Equine Distal Limbs: A Pilot Study Examining Jumping Exercise

**DOI:** 10.3390/ani9100751

**Published:** 2019-09-30

**Authors:** Steven Johnson, Jennifer Symons

**Affiliations:** Shiley School of Engineering, University of Portland, Portland, OR 97203, USA

**Keywords:** 3D scanning, horse, swelling, inflammation, exercise, jumping

## Abstract

**Simple Summary:**

When horse athletes exercise, forces are applied to their legs during each step. Repetitive loads applied during training and competition contribute to stress of leg structures. Swelling (i.e., local fluid accumulation) in the legs is a sign of increased stress in these structures. Currently, horse riders and trainers make training decisions based on visual observations of horse leg swelling, which are potentially subjective and imprecise. The aim of this study was to develop a low-cost, noninvasive method to measure lower leg volume in horses, as well as to assess the ability to detect differences before and after exercise. The tablet-mounted scanner measured increased lower leg volume after jumping, compared to scans before exercise. This method may serve as an objective tool for horse riders and trainers to prevent injury by modulating horse training frequency and intensity in response to physiological signs of stress, like leg swelling. Scans may also be used to seek veterinary consultation and treatment, as well as to guide rehabilitation from injury.

**Abstract:**

Equine athletes can incur musculoskeletal injuries due to repetitive loading during training and competition. Prior to signs of lameness, horse trainers and veterinarians may observe swelling in the distal limbs, where injuries most frequently occur. Early observations may guide modulation of training to manage physiological stress and mitigate risk of injury. However, these observations of changing limb volume can be subjective and imprecise. The aim of this study was to assess the accuracy and applicability of a tablet-mounted, 3D scanner to measure and record distal limb volumes of horses before and after exercise. Users recorded scans of a cylinder of known volume with errors up to 8%. Experienced users’ measures were biased (i.e., consistently overestimated). The scanner was able to detect statistically significant increases in volume for both fore and hind limbs after one jumping session (310–2058 cm^3^). Age and intensity of workload may play a role in magnitude of limb swelling, but had mixed conclusions between fore and hind limbs. More studies with additional horses must be performed to solidify these relationships. The evaluated 3D scanner is a low-cost, accessible tool that was able to detect changes in limb swelling as a result of exercise and mechanical stress. With continued research, this information may guide training programs to decrease injury and maximize performance of equine athletes in the future.

## 1. Introduction

Equine athletes undergo repetitive mechanical loading during training and competition. This cyclic loading can contribute to stress of musculoskeletal tissues in the limbs [1,2,3,4]. This fatigue contributes to injuries that occur in many different equestrian disciplines [5]. In the case of sport horses in dressage or showjumping, these injuries can compromise a horse’s competitive career and potential by disrupting training with layups and rehabilitation. In the case of racehorses, catastrophic musculoskeletal injuries (e.g., third metacarpal bone fracture, sesamoid bone fracture, or suspensory ligament rupture) may necessitate euthanasia. Epidemiological studies of racehorses have identified exercise history as a risk factors for these injuries [6,7]. Therefore, modulating training frequency and intensity have the potential to influence stress of musculoskeletal tissues and injury. However, equine athletes are part of a population that have variability in their response to mechanical stress. That is, two horses may respond differently to the same training program. Assessing each horse’s response individually is critical to tailoring training and mitigating injury. 

Symptoms are experienced by an affected individual, whereas signs are observed by someone other than the affected individual. Because horses cannot communicate symptoms of physiological stress, horse trainers and veterinarians make observations through sight and touch related to behaviors, movement patterns, and physiological signs of stress to musculoskeletal tissue. In advanced stages, these signs may include a lameness diagnosis resulting from initial veterinary workup that may be followed-up by various imaging modalities. Early physiological signs of mechanical stress include heat and swelling, primarily in the distal limbs where most musculoskeletal injuries occur in equine athletes [5,8]. This change in volume is a result of the body’s inflammatory response to increase fluid and white blood cell movement to the site. Unfortunately, visual observations can be subjective, imprecise, and inaccurate. An objective method to measure and record physiological signs in the distal equine limb may prove useful to inform training decisions and veterinary treatments. 

In humans, objective limb volume measurements have been used in sports to assess muscle hypertrophy in response to training [9], as well as in medicine to assess the efficacy of treatments to lessen edema [10]. Human medical and research studies have compared the strengths and weaknesses of several methods for objective limb volume measurements including perometry, water displacement, circumferential models, as well as computed tomography and magnetic resonance imaging [11,12]. Advanced imaging and perometry devices have the greatest accuracy and reproducibility, but are only available in specialized facilities due to their expense and size. Furthermore, these devices have U-shaped or closed ring arms that must travel around the measured limb, which can be difficult to use on equine patients [13]. Simpler methods like water displacement or circumferential models are cheaper and easier to deploy, but may not translate directly to the differing morphology and hair-covered surfaces of equine limbs. 

Advances in consumer electronics have provided solutions in previous scientific research. In particular, 3D scanners have been used in various medical applications, including mapping oral anatomy as it relates to airway obstruction [14] and measuring lymphedema in cancer patients [10]. In sport, this technology has been used to record anthropometric measurements previously assessed by advanced medical imaging [9]. Based on these previous successful applications, this technology may also prove to be useful in assessing changes in distal limb morphology of equine athletes. The objective of this study was to assess the accuracy and applicability of a 3D scanner as a low-cost (<$500), noninvasive tool to objectively measure distal limb volumes in horses. This research serves as a pilot study to assess equine athletes before and after jumping exercise. Horses were hypothesized to have greater limb volume after mechanical stress incurred while jumping, compared to scans before exercise. If proven useful, this method may be investigated for assessing horses in other disciplines or during rehabilitation from distal limb injuries, like the superficial digital flexor tendon. 

## 2. Materials and Methods 

All study protocols were approved by the University of Portland Institutional Animal Care and Use Committee (No. 2018-Symons-01). Five horses (13 ± 6 years old, Table 1) training and competing in showjumping activities participated in the study protocol with owner consent. All horses were assessed for general health (temperature, pulse, and respiration) by study researchers. Soundness and fitness to participate, as well as the intensity of jumping exercise between study scans, were determined by each horse’s owner, rider, and/or trainer. In some cases, riders and trainers may believe horses to be sound that are in fact clinically lame [15,16,17]. Horses in this study should be considered representative of a population of horses in weekly training programs that include lessons, training rides, and jumping, as well as competitions. 

All study scans were performed using a 3D scanner (Structure Scanner, Occipital, San Francisco, CA, USA) mounted to a tablet (iPad, Apple). The 3D scanner was calibrated using an associated, proprietary application. Calibration was achieved by slowly panning the tablet and attached 3D scanner to capture focal points on surrounding objects and surfaces, using both the camera on the tablet and the infrared camera within the 3D scanner. After calibration, the scanner may be used to map the surface of any object. Scans are recorded by videos from the embedded tablet camera and the mounted 3D scanner (up to 54 fps). When scanning, the tablet must travel along a circular path to view all sides of an object. Depending on the shape and dimensions of the object, the tablet may also need to record video at different orientations and heights. Each scan is a collection of vertices that represent the object’s surface. Typically, the distance between vertices is approximately 2 mm. 

Validation of the scanner was assessed by scanning an aluminum cylinder of known volume, which was covered with white paper to reduce scanning errors associated with reflective surfaces. In order to assess usability and accuracy, three individuals (two students, one faculty member within a lab) each performed three scans of the cylinder. After assessing the accuracy of the scanner, one student was selected to perform all subsequent horse scans to reduce interoperator errors.

All measurements and scans were performed in cross-ties at the horses’ primary stables. One fore limb and one hind limb was scanned from each horse. Limb sidedness (left or right) in fore and hind scans was assigned randomly (RAND, Microsoft Excel). Selected fore and hind limbs were scanned from the carpus/calcaneus to the hoof. Three replicate scans were performed for each selected fore and hind limb immediately prior to a jumping session, as well as 18 h after completing exercise. Timing of post-exercise scans was influenced by access to horses during stable hours, allowing maximum time for physiological response, and reducing likelihood of handwalks, turnouts, or additional exercise between scans. The jumping session was observed for duration, average jump height, and number of obstacles completed. Each of these exercise factors were selected by the rider, and were not influenced by the researchers or the study protocol. Jumping intensity was determined by thresholds for jump height (0.85 m) and number of obstacles (30). Jumping sessions exceeding both thresholds were rated as moderate intensity and jumping sessions below one or both thresholds were rated as low intensity.

The optimal distance between the scanner and the limb was 30–40 cm, as determined by experimental use. To allow for this optimal distance on all sides of the limb, particularly the medial aspect, an individual held the contralateral limb up, out of the view of the camera during each scan. This also helped prevent the horse from producing any large limb movements during scanning. Another individual moved the tablet and attached 3D scanner around all sides of the limb to create a digital mesh corresponding to the surface of the limb. In many cases, slow movements of the camera and/or revisiting previous views of the limb were used to minimize noise and scan errors. Each scan was completed in less than 3 min, with each scanning session lasting no more than 30 min.

Objects had to remain in the camera frame throughout the entire scanning process. This constraint was challenging when scanning the palmar surfaces of fore limbs and the dorsal surface of hind limbs. To avoid the need for individuals to travel under the horse’s trunk, a camera stand was used to hold the scanner steady under the trunk with continued view of the limb, while the operator safely moved to the other side of the horse, picked up the device, and continued scanning other surfaces of the limb. 

**Figure 1:**
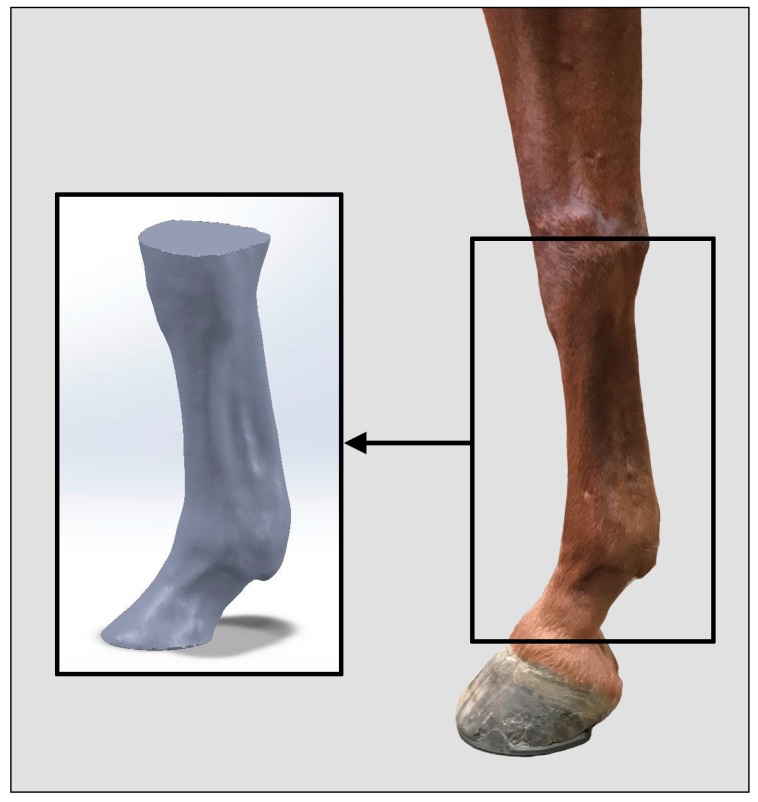
Processed equine distal fore limb mesh (left) relative to equine forelimb anatomy (right). All scans were cropped from the apex of the accessory carpal bone or calcaneus to the dorsal midline of the coronet band.

Scanned surfaces were saved as object files (OBJ) to allow for mesh processing (Fusion 360, Autodesk) that removed any noise attributed to neighboring surfaces other than the selected limb. Each mesh was scanned for discontinuity (i.e., water-tightness) and repaired if needed. Additionally, each shell was cropped with parallel planes at proximal (i.e., apex of accessory carpal bone or calcaneus) and distal (i.e., dorsal midline of coronet band) anatomical features (Figure 1). 

Processed meshes were saved as a stereolithography files (STL) to facilitate quantitative analyses in engineering computational software (MATLAB, Mathworks, Natick, MA, USA). Limb volumes were calculated using a convex hull function (convhull, MATLAB) that used the vertices in the STL file to return a volume using a Delaunay Triangulation method. 

User differences in measured cylinder volume were compared using a one-way ANOVA (PROC GLM, SAS). Horse limb volumes before and after jumping sessions were compared using a repeated measures ANOVA (PROC MIXED, SAS) with 3 factors: time of scan (i.e., before or after jumping), the interaction of time and jump intensity (i.e., height and number of obstacles), and the interaction of time and age as factors. In the mixed model, horse and all horse interaction terms were included as random factors. Normality of residuals was assessed visually within a QQ plot and determined to be acceptable.

## 3. Results

**Figure 2:**
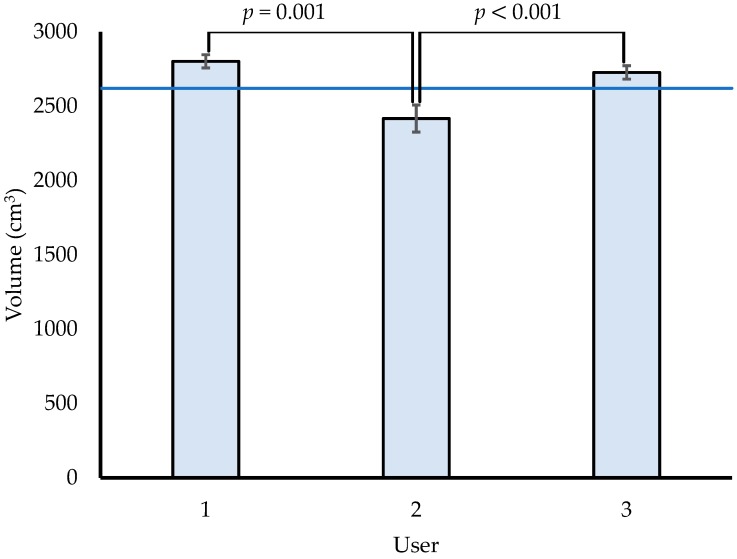
Interoperator scanning differences. Means and standard deviations for measured volumes of an aluminum cylinder from 3 users, compared to actual volume (blue line: 2620 cm^3^). User 2 volumes (2417 ± 91 cm^3^) were significantly different than volumes measured by Users 1 (2802 ± 44 cm^3^) and 3 (2727 ± 45 cm^3^). Users 1 and 3 were not significantly different and both consistently overestimated volumes (4%–7%).

The accuracy of a tablet-mounted 3D scanner was assessed, as well as the ability to detect changes in distal equine limb volume before and after jumping. All 3 users recorded mean volumes of a paper-covered, aluminum cylinder within 8% of the actual volume (Error = 106–203 cm^3^, Figure 2). User 2 recorded volumes significantly less than volumes recorded by users 1 and 3. Users 1 and 3 had substantially more experience using the scanner to measure many different objects, whereas user 2 had no prior experience using the scanner. Furthermore, Users 1 and 3 had less variability within measured volumes, compared to User 2. Measurements from experienced users (1 and 3) were biased, that is they consistently overestimated the volume of the cylinder (4%–7%). Measurements from User 2 were biased and consistently underestimated the volume of the cylinder (8%). User 1 performed all subsequent scans of horse limbs before and after jumping, based on their employment as a student researcher for this study. Within cylinder scans, this user had a bias of 7% overestimation. This bias was likely systematic and considered to be present in all subsequent limb scans to some degree.

Visual observations yielded no marked differences between left and right for any horses’ fore or hind limbs. All horses had larger distal fore and hind limb volumes after jumping, compared to initial scans (Table 2 and Table 3). With the exception of Horse 4, most changes in limb volume were apparent to an experienced observer familiar with distal limb anatomy. Most horses had changes in limb volumes of 4%–9% relative to initial volumes. However, the youngest horse (Horse 3) had changes in limb volume up to 37%. This horse’s rider disclosed that the observed session between scans involved markedly elevated exercise compared to the horse’s routine training. This elevated exercise was not influenced by the researchers and was not executed for experimental purposes. All participating riders were given full autonomy to determine each horse’s jumping activity. 

The evaluated workload was considered routine for nearly all study horses. Most observed changes in limb swelling were considered apparent, but unremarkable to riders and trainers. Therefore, these physiological signs did not warrant alteration in training frequency and intensity. In the case of Horse 3, the rider visually perceived the distal limb swelling also measured by the scanner and responded with decreased workload in the following week. However, the owner did not perceive any injuries warranting veterinary consult, treatment, lay-up, or rehabilitation. Therefore, the horse continued with routine training in the following weeks and months.

Within the ANOVA, time was significant for both fore and hind limbs. That is, post-exercise scans recorded larger distal limb volumes than pre-exercise scans. However, the interaction of time and age, as well as the interaction of time and intensity, were significant in fore limb scans, but not in hind limb scans (Table 4). Subsequent pair-wise comparisons revealed significant differences between scans before and after jumping for low and moderate sessions, as well as all sessions combined, in the fore limb (Table 5). In the hind limb, moderate sessions and all sessions combined had significant differences in limb volumes, whereas limb volume differences in low sessions were a statistical trend. Least square mean estimates of limb volume change ranged from 478 to 1084 cm^3^.

## 4. Discussion

A 3D scanner was assessed as a low-cost tool to objectively measure and record distal limb volumes in horses before and after exercise. The scanner was able to record volumes within 8% of the actual volume of a cylinder. The scanner was also able to detect a statistically significant increase in distal fore and hind limb volumes after jumping, compared to initial scans. 

3D scans and associated volumes are influenced by operator experience. Those users who had more initial practice using the device (Users 1 and 3) had less variability in recorded volumes, compared to a user with no prior experience. Furthermore, lack of experience using the scanner was also associated with underestimates of cylinder volume. Experienced users noted improved understanding of the relationship between camera position relative to the scanned object and visual feedback shown on the tablet screen. This feedback was particularly important for assessing scan quality (i.e., smoothness) and directing the lens toward any regions that required additional exposures to resolve roughness or misalignment of adjacent surfaces. Previous studies adopting this technology in other applications have not addressed needs for training or experience. However, some level of familiarity with the device and strategy for optimal distance/orientation during scanning seems to be beneficial in this setting. Differing factors in this application that may necessitate training could include environment and lighting outside of a traditional lab setting, as well as the shape and surface texture of limbs being scanned. Until inter-operator differences are fully understood, the same operator should perform all scans to assess a given horse. 

The magnitude of recorded limb volumes may not be considered accurate. Experienced users’ measurements were bias, with consistent, systematic errors that overestimated the actual cylinder volume. Users 1 and 3 recorded mean volumes up to 181 cm^3^ larger than the actual cylinder volume (2620 cm^3^). These inaccuracies were biased when measuring an object with a smooth, matte surface. Therefore, the magnitude of a volume associated with an individual scan of a horse’s leg that is covered with hair and unique curvatures may be considered equally or more inaccurate. 

Despite measurement inaccuracies, observed differences in limb volume may be considered significant. Errors that contribute to overestimated volumes are likely consistent across all measurements of similar surfaces performed with the same scanner. Furthermore, statistical analyses confirmed differences in limb volume before and after exercise were consistently larger than variability of scans within a session (i.e., signal to noise ratio). Because limb volume is not expected to change within a scanning session, variability of scans within a session reflects variability of the operator and scanner combined. Therefore, differences observed between conditions remain significantly different relative to any variability in the operator and/or scanner. However, the magnitude of these differences may be inaccurate and is likely overestimated. That is, the observed change in volume may not directly reflect additional fluid within the limb. 

Greater jump intensity did not consistently contribute to greater limb swelling. Least square means indicate greater changes in forelimbs under low intensity jumping and hindlimbs under moderate intensity jumping. These results are inconsistent with prior studies. Increases in fence height have been shown to increase forelimb loads within the superficial digital flexor tendon [18]. Therefore, distal structures are expected to undergo greater mechanical stresses over larger fences that should result in greater physiological responses like limb swelling. Similarly, muscles and tendons in the hindlimbs are responsible for producing greater energy in jumping than the forelimbs [19], which should correspond to increased loading and limb swelling. However, the magnitude of observed changes in limb volume don’t align with these prior studies in kinetics. These discrepancies are likely attributed to a small sample size in the current study, as well as horses having variability in conformation, training history, prior injury, and unknown physiological factors that may affect their responses to mechanical stress. Additional scans from a greater population of horses are needed to confirm this method. However, these varied responses support the need for methods of individual assessment of equine athletes that may have unique physiological responses that are inconsistent with mechanics theory.

Horse age may affect the magnitude of limb swelling that occurs after exercise. The youngest observed horse had the greatest changes in limb volume after jumping. It is unclear if this marked change is attributed to the horse’s age or other factors. However, some studies have observed increased responses to physiological stress in young animals, compared to older animals [20,21]. Increased sensitivity to mechanical stress in young animals may warrant increased monitoring of physiological responses like swelling to modulate training. Older animals may be less sensitive in their physiological response to changes in exercise. Future studies may seek to observe more horses of varying ages to investigate this potential relationship between tissue stress, swelling and age. 

The results and conclusions of this pilot study are limited by the small sample size and limited range of jump intensity executed by subjects. Small sample sizes may be particularly misleading when considering factors that further divided the 5 subjects observed (i.e., one young horse, or 2–3 horses per jump intensity group). Within these smaller samples, each individual horse bears a large influence in the statistical model’s estimates. Future studies may seek to evaluate more horses over a wider range of ages and jump heights to solidify hypothesized relationships between these factors and limb swelling. Increased knowledge and understanding of these relationships may prove useful in modulating training frequency and intensity to mitigate risk of injury and enhance performance in equine athletes. 

## 5. Conclusions

The evaluated 3D scanner was able to detect differences in limb swelling before and after jumping in horses with varied ages and workloads. This methodology may prove useful in assessing physiological responses to elevated, non-routine mechanical stresses. Future studies may also investigate the use of this technology in other disciplines or during rehabilitation from distal limb injuries, particularly superficial soft tissue structures like suspensory branches or the superficial digital flexor tendon. With continued scientific evaluation, this technology may provide information to guide training and rehabilitation programs to lessen the incidence of injury and maximize potential performance of equine athletes in the future. 

## Figures and Tables

**Table 1:** Background of horse subjects. Jump intensity was recorded categorically (moderate or low) and quantitatively (jump height, number of obstacles).

Horse	Sex	Breed	Age (years)	Height (m)	Jump Intensity
1	Gelding	Warmblood	18	1.73	Moderate (1.10 m, 35)
2	Gelding	Warmblood	10	1.85	Moderate (1.10 m, 37)
3	Mare	Warmblood Cross	4	1.68	Moderate (0.85 m, 35)
4	Gelding	Draft Cross	16	1.65	Low (0.7 m, 14)
5	Gelding	Thoroughbred	16	1.75	Low (0.9 m, 23)

**Table 2:** Forelimb volume of horse subjects before and after jumping (*n* = 3 scans per horse before exercise and after exercise, Mean ± SD). All horses had varied increases in limb volume 18 h after jumping exercise, compared to initial scans.

Horse	Volume Before (cm^3^)	Volume After (cm^3^)	Difference (cm^3^)	Percentage
1	7872 ± 92	8288 ± 132	416	5%
2	6716 ± 285	7340 ± 135	624	9%
3	5633 ± 478	7691 ± 167	2058	37%
4	5591 ± 162	5911 ± 51	320	6%
5	5344 ± 633	6398 ± 1625	1054	20%

**Table 3:** Hindlimb volume of horse subjects before and after jumping (*n* = 3 scans per horse before exercise and after exercise, Mean ± SD). All horses had varied increases in limb volume 18 h after jumping exercise, compared to initial scans.

Horse	Volume Before (cm^3^)	Volume After (cm^3^)	Difference (cm^3^)	Percentage
1	5889 ± 202	6339 ± 145	450	8%
2	10,667 ± 283	11,215 ± 75	548	5%
3	3599 ± 140	4418 ± 347	819	23%
4	8565 ± 686	8875 ± 407	310	4%
5	8083 ± 434	8564 ± 744	481	6%

**Table 4:** Type 3 tests of fixed effects. Time of scans (i.e., before or after jumping) was significant in fore and hind limbs, whereas the interaction of age and jumping intensity with time had mixed conclusions.

Factor	Forelimb		Hindlimb	
	F-Value	*p*-Value	F-Value	*p*-Value
Time	15.941	<0.001	12.144	0.002
Time × Age	11.601	<0.001	0.470	0.68
Time × Intensity	29.420	<0.001	0.030	0.971

**Table 5:** Least square means and standard errors for limb volumes before and after jumping, as well as differences. Least square means and standard errors represent an aggregate of all study horses, adjusted to the mean age (i.e., 13 years old).

Limb	Intensity	Volume Before (cm^3^)	Volume After (cm^3^)	Difference (cm^3^)	*p*-Value
Fore	Low	4916 ± 288	6000 ± 267	944 ± 229	0.011
	Moderate	7062 ± 209	7867 ± 209	805 ± 296	0.012
	Combined	5989 ± 167	6934 ± 158	1084 ± 393	<0.001
Hind	Low	9024 ± 1969	9501 ± 1969	478 ± 248	0.067
	Moderate	8656 ± 1766	9207 ± 1766	551 ± 197	0.010
	Combined	8840 ± 1178	9354 ± 1178	514 ± 147	0.002

## References

[1] Biewener A.A., Thomason J., Goodship A., Lanyon L.E. (1983). Bone stress in the horse forelimb during locomotion at different gaits: A comparison of two experimental methods. J. Biomech..

[2] Crevier-Denoix N., Pourcelot P., Ravary B., Robin D., Falala S., Uzel S., Grison A.C., Valette J.P., Denoix J.M., Chateau H. (2009). Influence of track surface on the equine superficial digital flexor tendon loading in two horses at high speed trot. Equine Vet. J..

[3] Martig S., Chen W., Lee P.V.S., Whitton R.C. (2014). Bone fatigue and its implications for injuries in racehorses. Equine Vet. J..

[4] Morrice-West A.V., Hitchens P.L., Walmsley E.A., Stevenson M.A., Whitton R.C. (2019). Training practices, speed and distances undertaken by Thoroughbred racehorses in Victoria, Australia. Equine Vet. J..

[5] Murray R.C., Dyson S.J., Tranquille C., Adams V. (2006). Association of type of sport and performance level with anatomical site of orthopaedic injury diagnosis. Equine Vet. J..

[6] Hitchens P.L., Morrice-West A.V., Stevenson M.A., Whitton R.C. (2019). Meta-analysis of risk factors for racehorse catastrophic musculoskeletal injury in flat racing. Vet. J..

[7] Hitchens P.L., Hill A.E., Stover S.M. (2018). Relationship Between Historical Lameness, Medication Usage, Surgery, and Exercise With Catastrophic Musculoskeletal Injury in Racehorses. Front. Vet. Sci..

[8] Estberg L., Stover S.M., Gardner I.A., Johnson B.J., Case J.T., Ardans A., Read D.H., Anderson M.L., Barr B.C., Daft B.M. (1996). Fatal musculoskeletal injuries incurred during racing and training in thoroughbreds. J. Am. Vet. Med. Assoc..

[9] Kordi M., Haralabidis N., Huby M., Barratt P.R., Howatson G., Wheat J.S. (2019). Reliability and validity of depth camera 3D scanning to determine thigh volume. J. Sports Sci..

[10] Öhberg F., Zachrisson A., Holmner-Rocklöv Å. (2014). Three-Dimensional Camera System for Measuring Arm Volume in Women with Lymphedema Following Breast Cancer Treatment. Lymphat. Res. Biol..

[11] Sharkey A.R., King S.W., Kuo R.Y., Bickerton S.B., Ramsden A.J., Furniss D. (2018). Measuring Limb Volume: Accuracy and Reliability of Tape Measurement Versus Perometer Measurement. Lymphat. Res. Biol..

[12] Batista B.N., Baiocchi J.M.T., Campanholi L.L., Bergmann A., Duprat J.P. (2018). Agreement between Perometry and Sequential Arm Circumference Measurements in Objective Determination of Arm Volume. J. Reconstr. Microsurg..

[13] Haase F., Siewert C., von Rautenfeld D.B., Fischbach J.U., Seifert H. (2009). Comparison of different methods to quantify the volume of horse limbs. Berl. Munch. Tierarztl. Wochenschr..

[14] Das A.J., Valdez T.A., Vargas J.A., Saksupapchon P., Rachapudi P., Ge Z., Estrada J.C., Raskar R. (2016). Volume estimation of tonsil phantoms using an oral camera with 3D imaging. Biomed. Opt. Express.

[15] Greve L., Dyson S.J. (2014). The interrelationship of lameness, saddle slip and back shape in the general sports horse population. Equine Vet. J..

[16] Rhodin M., Roepstorff L., French A., Keegan K.G., Pfau T., Egenvall A. (2016). Head and pelvic movement asymmetry during lungeing in horses with symmetrical movement on the straight. Equine Vet. J..

[17] Pfau T., Jennings C., Mitchell H., Olsen E., Walker A., Egenvall A., Tröster S., Weller R., Rhodin M. (2016). Lungeing on hard and soft surfaces: Movement symmetry of trotting horses considered sound by their owners. Equine Vet. J..

[18] Meershoek L.S., Schamhardt H.C., Roepstorff L., Johnston C. (2001). Forelimb tendon loading during jump landings and the influence of fence height. Equine Vet. J. Suppl..

[19] Bobbert M.F., Santamaría S. (2005). Contribution of the forelimbs and hindlimbs of the horse to mechanical energy changes in jumping. J. Exp. Biol..

[20] Cunningham H.C., West D.W.D., Baehr L.M., Tarke F.D., Baar K., Bodine S.C., Christiansen B.A. (2018). Age-dependent bone loss and recovery during hindlimb unloading and subsequent reloading in rats. BMC Musculoskelet. Disord..

[21] Holguin N., Brodt M.D., Sanchez M.E., Silva M.J. (2014). Aging diminishes lamellar and woven bone formation induced by tibial compression in adult C57BL/6. Bone.

